# Deposition of Cellulose-Based Thin Films on Flexible Substrates

**DOI:** 10.3390/ma11122433

**Published:** 2018-11-30

**Authors:** Werner Schlemmer, Armin Zankel, Katrin Niegelhell, Mathias Hobisch, Michael Süssenbacher, Krisztina Zajki-Zechmeister, Michael Weissl, David Reishofer, Harald Plank, Stefan Spirk

**Affiliations:** 1 Institute for Paper-, Pulp- and Fibre Technology, Graz University of Technology, Inffeldgasse 23, 8010 Graz, Austria; werner.schlemmer@tugraz.at (W.S.); K.niegelhell@gmx.at (K.N.); mathias.hobisch@tugraz.at (M.H.); michael.suessenbacher@student.tugraz.at (M.S.); michael.weissl@tugraz.at (M.W.); david.reishofer@anton-paar.com (D.R.); 2 Institute of Electron Microscopy and Nanoanalysis (FELMI), Steyrergasse 17, 8010 Graz, Austria; armin.zankel@felmi-zfe.at (A.Z.); krisztina.zechmeister@felmi-zfe.at (K.Z.-Z.); harald.plank@tugraz.at (H.P.)

**Keywords:** cellulose, thin films, trimethylsilyl cellulose, superhydrophobic, metal foils

## Abstract

This study investigates flexible (polyamide 6.6 PA-6.6, polyethylene terephthalate PET, Cu, Al, and Ni foils) and, for comparison, stiff substrates (silicon wafers and glass) differing in, for example, in surface free energy and surface roughness and their ability to host cellulose-based thin films. Trimethylsilyl cellulose (TMSC), a hydrophobic acid-labile cellulose derivative, was deposited on these substrates and subjected to spin coating. For all the synthetic polymer and metal substrates, rather homogenous films were obtained, where the thickness and the roughness of the films correlated with the substrate roughness and its surface free energy. A particular case was the TMSC layer on the copper foil, which exhibited superhydrophobicity caused by the microstructuring of the copper substrate. After the investigation of TMSC film formation, the conversion to cellulose using acidic vapors of HCl was attempted. While for the polymer foils, as well as for glass and silicon, rather homogenous and smooth cellulose films were obtained, for the metal foils, there is a competing reaction between the formation of metal chlorides and the generation of cellulose. We observed particles corresponding to the metal chlorides, while we could not detect any cellulose thin films after HCl treatment of the metal foils as proven by cross-section imaging using scanning electron microscopy (SEM).

## 1. Introduction

In industry, the modification of different types of surfaces is a crucial aspect in materials design and development. Particularly, the interaction of polymer thin films with other surfaces is a major issue in the development of engineered composite/hybrid materials and interfaces [1,2,3]. Cellulose, the major biopolymer on earth, has not been extensively investigated in this context [4,5,6,7,8]. This originates from the inherent insolubility of cellulose in common organic solvents, which often makes direct processing into thin films tedious [9,10,11,12]. The most convenient route to manufacturing cellulose thin films is the use of soluble precursors which are regenerated back after film deposition [13,14]. Here, trimethylsilyl cellulose, a cellulose ether whose solubility can be tuned over a wide range by the degree of substitution with silyl groups, is the most favorable choice for several reasons [14,15,16]. At high degree of substitution with trimethylsilyl groups (DS_TMS_), TMSC has good solubility in organic solvents (e.g., CHCl_3_) and homogenous films can be produced by spin coating or Langmuir Schaefer deposition [14,17]. After the processing steps, these TMSC films can be converted into pure cellulose by exposure to HCl vapors [16]. So far, in most of the previous studies, the focus was to deposit the films on flat, stiff, and rather small (1 cm × 1 cm) substrates, such as silicon wafers, leading to the formation of uniform cellulose thin films. These confined two-dimensional films have been employed to unravel the interaction with other biopolymers, such as proteins and polysaccharides [18,19], as well as to gain a deeper understanding of water-cellulose interactions with respect to cell-wall hierarchy and industrial drying technologies, since they are mostly of amorphous nature [20,21,22,23]. However, the films can also be used as functional layers in optoelectronic devices, such as thin film transistors [24,25] and photovoltaics [26].

In order to extend the application range of cellulose thin films to other areas, a systematic approach to explore their film formation properties as well as their influence on surface properties is required. Just a few substrates have been reported for cellulose thin film deposition, such as silicon wafers [14], glass [27], mica [28], gold [29], and CaF_2_ [30]. In this study, we explore various substrates which impose challenges in the preparation of cellulose thin films due to the topology, surface free energy, chemical sensitivity towards the regeneration procedure, and flexibility/bendability of the substrate. We also elaborate on the coating of larger substrates than those reported in the literature so far. 

The paper is constructed as follows: in the first part, the formation and characterization of TMSC films on the different substrates is studied, while in the second part, their conversion to cellulose is attempted and followed. 

## 2. Materials and Methods

TMSC (Avicel, M_w_ = 185,000 g/mol, M_n_ = 30,400 g/mol, polydispersity index = 6.1 determined by size exclusion chromatography in chloroform) with a DS_TMS_ value of 2.8 was purchased from TITK (Rudolstadt, Germany). Chloroform (99.3%) and acetone (99.7%) were purchased from Sigma-Aldrich (St. Louis, MO, USA) and Brenntag CEE GmbH (Vienna, Austria). Hydrochloric acid (37%) and sulfuric acid (95%) were obtained from VWR Chemicals (Radnor, PA, USA). 2-Propanol was obtained from Fisher Scientific (Hampton, NH, USA). For the contact angle measurements, Milli-Q water (resistivity = 18.3 Ω^−1^·cm^−1^) and diiodomethane were used (Sigma Aldrich, 99%). All chemicals were used without further purification.

The polymer foils Europe 100 economy overhead transparencies were purchased from Avery Zweckform (Oberlaindern, Germany), and PA-6.6 cast film monolayer foils were supplied by BASF SE (Ludwigshafen, Germany). The microstructured copper foil was kindly provided by AT&S (Leoben, Austria), and a nickel foil with a thickness of 40 µm was used. Chromafil Xtra syringe filters made from polyvinylidene difluoride (PVDF)-45/25 0.45 μm pore size (Macherey Nagel, Düren, Germany) were used as obtained.

### 2.1. Substrate Cleaning and Film Preparation

Prior to spin coating, glass slides (7.3 × 10 cm²) and silicon wafers (2 × 1 cm²) were immersed in a “piranha” solution containing H_2_O_2_ (30 wt%) and H_2_SO_4_ (1:3 v/v) for 10 min, then extensively rinsed with deionized water and blow-dried with N_2_ gas. The PET and PA-6.6 foils were first wiped with acetone and then immersed in an isopropanol bath of a VWR ultrasonic cleaner (VWR, Radnor, PA, USA) with 60 Hz equipped with an integrated heater. The other substrates (Cu, Ni, Al (7.3 × 10, 2 × 1, 2 × 1 cm²)) were used without further purification steps.

The TMSC films were prepared by deposition of TMSC (1 wt% in chloroform) onto the substrates followed by spin coating (4000 rpm rotation speed, 2500 rpm·s^−1^ acceleration, 60 s). Regeneration was carried out by exposing the samples to HCl vapor until the characteristic Si–C band at 1252 cm^−1^ vanished (30 min for Cu, Al, Ni; 20 min for PET; 12 min for glass and silicon). While the size of the samples used for the atomic force microscopy (AFM) measurements had a size of 0.7 × 0.7 cm^2^ (70 µL TMSC solution was applied), for the other analyses, 1 × 2 cm^2^ substrates were prepared (280 µL TMSC solution was applied).

**Figure 1:**
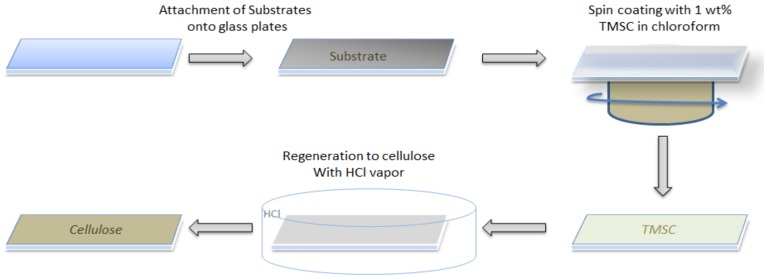
Workflow of the coating and regeneration of thin films on different substrates.

In order to avoid bending of the polymer and metal foils during the spin coating, the substrates were attached to glass plates of the same size using a double-faced adhesive tape. For further analyses, these samples were carefully removed from the plate after the coating procedure by tweezers. The general procedure is depicted in Figure 1.

### 2.2. Stylus Profilometry

A Dektak XT stylus profilometer (Bruker, Billerica, MA, USA) was used with Vision64 1-1-4-4 software (Bruker) to process the data. For each measurement, 1000 µm of the sample were scanned in 10 s with a resolution of 0.33 µm/pt. and an amount of 3001 points per sample. The stylus radius was 12.5 µm, which was pressed on the samples of 3 mg weight using a standard scan method and a measurement range of 6.5 µm. The samples were scanned after scratching the films, and all measurements were performed in four parallels.

### 2.3. Contact Angle Measurements and Surface Free Energy

The static contact angle measurements were performed by a Drop Shape Analysis System DSA100 (Krüss GmbH, Hamburg, Germany) with a T1E CCD video camera (25 fps) and the DSA1 v 1.90 software (Krüss GmbH). Measurements were done using Milli-Q water and diiodomethane, using a droplet size of 3 μL and a dispense rate of 400 μL·min^−1^. All measurements were performed at least five times on two equivalent samples per experiment. Static contact angles (SCA) were calculated with the Young−Laplace equation, and the surface free energy (SFE) was determined with the Owen–Wendt–Rabel–Kaelble (OWRK) method [31,32,33]. In order to determine the water contact angle hysteresis, a drop of 6 µL Milli-Q water was placed on the substrates and the size was slowly increased in 0.5 µL steps until the advancing contact angle was maintained constant (maximum 17.5 µL). Subsequently, the volume was decreased again by applying the same step size until the plateau of the receding angle was reached. The dynamic water contact angles were determined as the mean value of the advancing and receding contact angles. Measurements were done in a laboratory which had standardized conditions (22 °C, 50% relative humidity).

### 2.4. Infrared Spectroscopy

IR spectra were acquired on an Alpha fourier-transform infrared spectroscopy (FTIR) spectrometer (Bruker, Billerica, MA, USA) using an ALPHA’s Platinum ATR single reflection diamond ATR module. Spectra were measured in a scan range between 4000 and 400 cm^−1^, forming an average over 48 scans and a resolution of 4 cm^−1^. The data was analyzed with the OPUS 4.0 software (Bruker) and normalized at 1100 cm^−1^ to create difference spectra.

### 2.5. Light Microscopy

An Olympus BX60F5 microscope (Olympus, Shinjuku, Japan) equipped with an Olympus e 520 reflex camera (Olympus) was used to image the coated metal foils. The pictures were obtained with a magnification of 1:500 in reflective light mode. The processing was carried out with an open-access image processing software (Gimp 2.10.2).

### 2.6. Atomic Force Microscopy Measurements

Surface morphology and roughness of the films on Si, glass, Cu, Ni and Al were determined on a Veeco Multimode Quadrax MM scanning probe microscope (Bruker, Billerica, MA, USA) using Si cantilevers (NCH-VS1-W from NanoWorld AG, Neuchatel, Switzerland) with a resonance frequency of 320 kHz and a force constant of 42 N·m^−1^. On the polymer substrates, the films were analyzed using a FastScanBio AFM using a FastScan-A cantilever (both from Bruker NANO) with typical resonance frequencies and force constants around 1.4 MHz and 18 N·m^−1^, respectively. All samples were analyzed in tapping mode in an ambient atmosphere at room temperature at the lowest possible force load and scan rates were adapted to obtain reliable surface data. Root mean square (RMS; R_q_) roughness calculation and image processing was performed with the Nanoscope software (V7.30r1sr3, Veeco, Plainview, NY, USA).

### 2.7. Scanning Electron Microscopy

The films deposited on copper, aluminum, and nickel foils were embedded in epoxy resin (Buehler GmbH, Braunschweig, Germany) and cut using an Ultramicrotome UC6 (Leica Microsystems, Vienna, Austria) equipped with a Histo Diamondknife 45° (Diatome AG, Nidau, Switzerland). They were consequently steamed with carbon and investigated using a ZEISS Sigma 300 VP electron microscope (Carl Zeiss AG, Oberkochen, Germany) equipped with an Everhart–Thornley detector (SE2) for the detection of secondary electrons. The film thickness of selected samples was determined at 18 points per sample using an open-access software and then statistically evaluated. Additionally, elemental analysis was performed using an SDD detector (OXFORD, Oxford, England) for energy-dispersive X-ray spectroscopy (EDX). To obtain the distribution of different chemical elements, EDX was performed at acceleration voltages of 7 (Al) and 15 kV (Cu, Ni).

## 3. Results

The first challenge to overcome in the modification of metal foils and polymer films featuring several micrometers of thickness is their intrinsic flexibility, which principally impedes the formation of homogenous films by spin coating; a phenomenon that scales with the size of the substrates. These problems originate from the vacuum which is applied to fix the substrate on the rotating disk during spin coating. A straightforward way to tackle this is to employ a rigid carrier for the substrates, which was a glass plate in our case. The substrates were adhered to the glass plates by sticky tape. Although in principle, any type of sticky tape can be used, a poorly adhering one facilitates the removal of the substrates from the rigid carrier once spin coating has been accomplished. After mounting the foils and films onto the glass plates, TMSC was deposited and subjected to spin coating. For all the films, homogeneous coatings were obtained, but with subtle differences in film thickness and appearance due to their differently rough surfaces. While homogeneous coatings could be applied onto the nickel and aluminum foils (R_q_ values of the TMSC film were approximately 10 and 40 nm, compared to the native Al and Ni R_q_ values of 17 and 40 nm, respectively), for the copper foil, observation of the film formation was difficult using AFM. The TMSC layer adheres to the microscopically rough surface, and hardly any reduction in the R_q_ is determined by AFM (neat Cu: 507 nm, TMSC coating on Cu: 500 nm). For the SiO_2_-based substrates and the polymer foils, the thin films feature a thickness between 125 and 140 nm, as determined by profilometry. For the other materials, profilometry did not yield any useful results, since the roughness of the films was very large, leading to high standard deviations. Therefore, for the films on the metal foils, the materials were embedded into epoxy resin and the cross section was analyzed by SEM. These images clearly reveal the TMSC layers on the metal substrates (see morphology section) with film thicknesses ranging from approximately 100 to 1600 nm (Table 1). 

Exposure to HCl vapors led to significant shrinkage of the films, caused by the removal of bulky trimethylsilyl (TMS) groups from the films concomitantly with an increase in density due to the formation of hydrogen bonds in cellulose. Interestingly, the required time for regeneration varied between the substrates, as proven by ATR-IR spectroscopy. The typical IR bands for the TMS group (1250 δ(Si–C); 850 ν(Si–O); 750 cm^−1^ ν(Si–C); see Supplementary Materials, Figure S1) were used to monitor the regeneration process [34]. While on the polymer, glass, and silicon samples, regeneration was completed after 15 min, it took around 30 min until the characteristic bands disappeared on the metal foils [35]. It is known that reactions involving gases are diffusion-limited, according to the Flory Huggins theory. Therefore, thicker films require a longer time period to be fully regenerated, since the length of the diffusion path of the reactant and the side product (i.e., TMS–Cl in our case) increases. In general, conversion is believed to start at the surface of the films before the ‘bulk’ gets converted. More details on the kinetics of TMSC conversion to cellulose have already been presented [36,37,38]. TMSC powder, for instance, requires up to 100 min until regeneration is completed using HCl vapors. A complication arose for the analysis of the metal foils, in that the HCl vapor seemed to corrode the surface (due to formation of metal chlorides, oxides), making the identification of cellulose very challenging. We approached this challenge by acquiring SEM-EDX images to identify the presence of the chlorides (see SEM section).

To visualize the homogeneity of the coatings on the metal substrates, light microscopy images (Supplementary Materials, Figure S2) revealed the grooved morphology of the Al and Ni foils. Coating the substrates with TMSC leads to smoother substrates, where the polymer fills the grooves of Al and Ni and seems to form a uniform coating. Upon regeneration, the shrinkage of the coating is visible.

### 3.1. Surface Morphology—Atomic Force Microscopy

**Figure 2:**
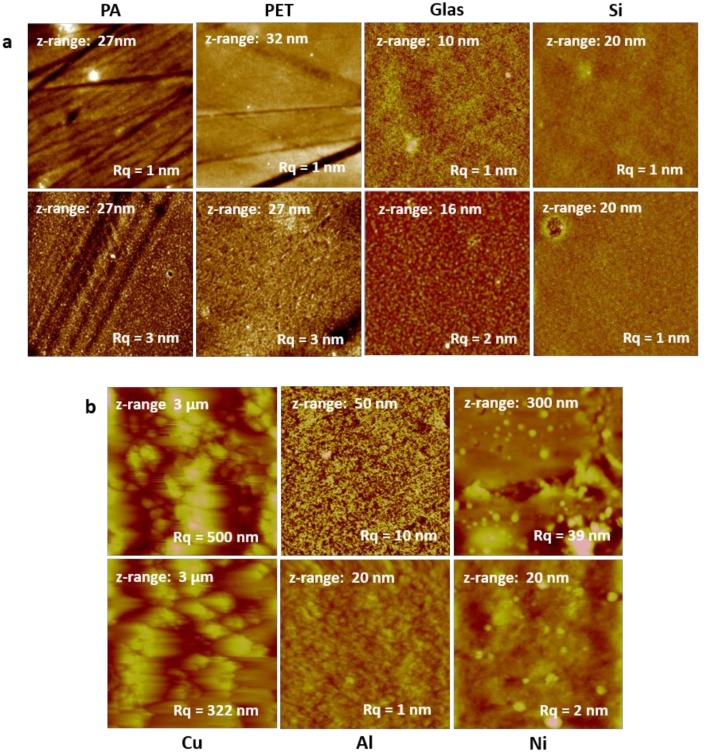
Atomic force microscopy (AFM) topography images (10 × 10 µm^2^) of TMSC layers before (upper row) and after exposure to HCl vapors (lower row) deposited on (**a**) PA, PET, glass, and silicon; and (**b**) Cu, Al, and Ni foils.

AFM topography images of the TMSC layers deposited on glass, silicon, PET, and PA are summarized in Figure 2a. It can be clearly seen that on the flat SiO_2_-based substrates, homogenous TMSC films were obtained, which did not significantly change their morphology after HCl exposure. In contrast, the neat polymer foils are a bit uneven and feature some scratchy defects. These scratches were everywhere on the investigated specimens, regardless of the cleaning procedure or handling. It is probable that these are already formed as a result of the manufacturing process. However, the TMSC adheres very well on these substrates, and homogenous layers with low roughness (R_q_ approximately 1 nm) were obtained, which only slightly increased after regeneration to cellulose (R_q_ approximately 3 nm). 

For the metal foils, the roughness of the substrate is more or less copied into the film structure. Depending on the metal foil used, the R_q_ ranged from 507 nm for copper down to 13 nm for the nickel foil (Figure 2b). On nickel and aluminum, TMSC forms quite homogeneous films, while on copper, it is difficult to judge on the basis of AFM measurements, since the roughness of the substrate is very high.

The exposure of the TMSC-coated metal foils to HCl vapor leads to the formation of grains on the surface, which is most pronounced for the nickel substrate, but also visible for the other two materials. Probably, the HCl vapor causes the formation of metal chlorides, which are directly formed at the TMSC/cellulose metal interface. This will be discussed in detail in the SEM section.

### 3.2. Scanning Electron Microscopy

**Figure 3:**
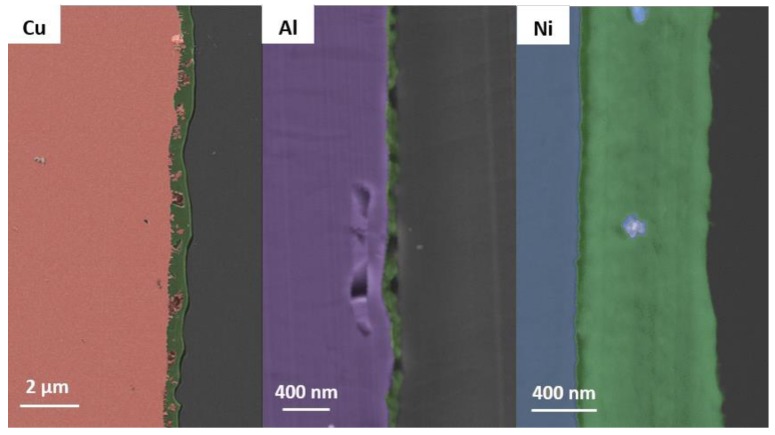
SEM images of cross sections of TMSC layers (green) deposited on Cu (red, left), Al (purple, middle), Ni (blue, right) foils. Before microtomy the samples were embedded in a resin (grey).

In order to get more insights into the film formation of TMSC at the interfaces between the metal foils, SEM images of the cross sections were acquired (Figure 3). The specimen preparation involved embedding of the substrates in epoxy resin, followed by microtomy. The morphology of these slices was then observed in SEM, wherein a complication arises due to the sensitivity of TMSC in an electron beam, which can easily lead to the regeneration of the TMSC, as reported earlier (Figures S3 and S4) [39]. 

The SEM images confirm most of the abovementioned hypotheses. However, for the rough materials such as the copper foil, the images clearly show that there is a homogeneous film deposited on the copper foil. Nevertheless, as already seen in the AFM images, the roughness is quite high and shows strong variations over the investigated sample area. For the nickel and aluminum foils, the homogeneity of the metal interface is much higher and therefore a homogenous coating can be deposited onto the surface. Yet, as mentioned before, it turned out that during the measurements, the TMSC already started to regenerate into cellulose, which can be seen by the holes present in the aluminum cross-section image. More images on nickel foil and how the acceleration voltage influences the TMSC can be found in the Supplementary Materials. Upon exposure of the TMSC on the metal foils to acidic conditions, particles are formed at the interface with the cellulose films, which consist of metal chlorides and oxides. It is known that metal oxides present on the metal surfaces can be easily converted to the corresponding chlorides when treated with HCl. The particles are relatively large, and it was not possible to identify a homogenous cellulose layer in those samples by SEM (Supplementary Materials, Figure S5). Elemental mapping by EDX (Supplementary Materials, Figure S6) confirmed the presence of metal chlorides and oxides in the HCl exposed films. However, there was also a significant amount of carbon detected, which means that cellulose is also present on these surfaces. 

### 3.3. Wettability of TMSC Coatings with Water

Static contact angle (SCA) measurements do not account for the volume dependency of the CA. Thus, in order to comprehensively describe the wettability, contact angle hysteresis (CAH) measurements were carried out. Especially when investigating the wettability of substrates with surface structuring in the centimeter to micrometer scale, the CAH measurement is a reliable tool for surface characterization. For this purpose, a drop was placed on the surface and the volume was slowly increased while monitoring the contact angle (see the Supplementary Materials). The angle increases until a plateau is reached: the so-called advancing contact angle (ACA). Upon the subsequent reduction of the volume, a similar behavior can be observed, leading to obtaining the receding contact angle (RCA) [40]. In many cases, the SCA is the average of the ACA and the RCA (cos θ_SCA_ = (cosθ_ACA_ + cosθ_RCA_)/2), although a general equation to derive this does not exist. The determination of the CAH of cellulosic surfaces is particularly challenging. Cellulose strongly interacts with water (e.g., via the formation of hydrogen bonds), which results in swelling of the substrate. Partial oxidation of cellulose at the surface (which is not the case in our samples) may even promote swelling [41]. Thus, the drop size as well as the moment of the CA measurement has a strong influence on the result. To obtain comparable values, the angles were determined 5 s after each drop size increase/decrease, and the resulting curves are shown in the Supplementary Materials. 

**Figure 4:**
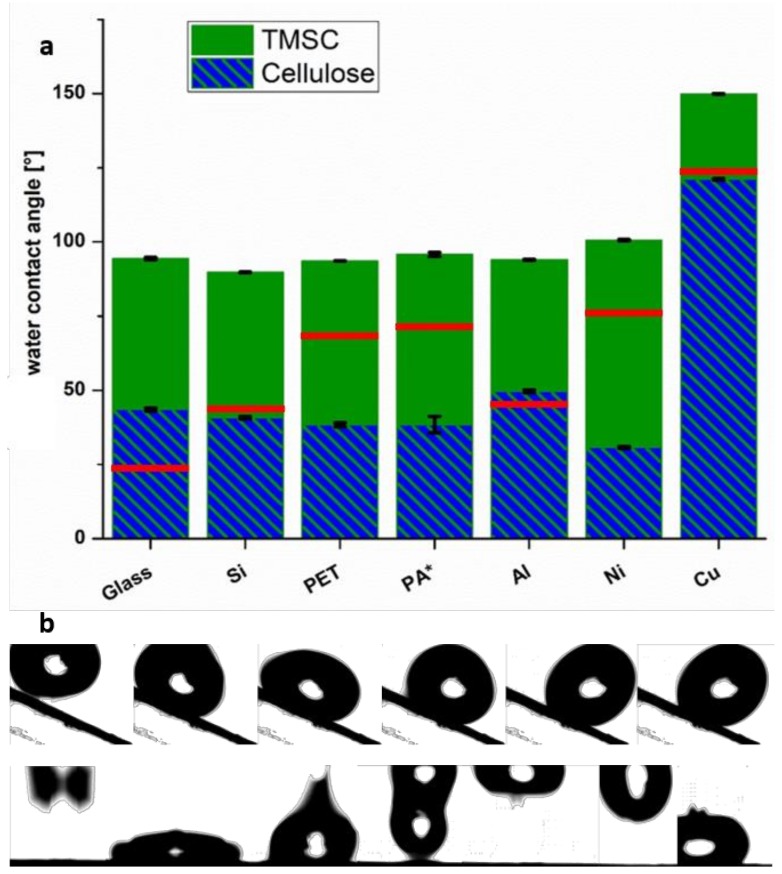
(**a**) Dynamic water contact angles of TMSC-coated substrates before (green) and after (blue) regeneration as well as the blank values (red lines). Due to the curved textures of PA, only the SCA could be determined. (**b**) Outtakes from a video of water drops (100 µL) rolling down a tilted (upper row) and bouncing off a flat (lower row) superhydrophobic surface (Cu–TMSC).

The sterically demanding, nonpolar silyl groups show strongly repulsive interactions with the polar water, leading to high water contact angles on all the TMSC-coated substrates. TMSC coatings on glass, silicon, PET, and PA feature nearly identical water contact angles of approximately 100°, while on aluminum and nickel, angles of 101° and 118° were determined, respectively (Figure 4a). The TMSC deposited on copper changed the wetting properties in a remarkable way: unexpectedly, superhydrophobicity was observed (θ = 153°) [42]. Superhydrophobic surfaces are defined by a water contact angle which exceeds 150°. This can be explained considering the equations developed by Cassie, Baxter, and Wenzel [43,44,45], which describe the change of the contact angle between air, liquids, and solids with increasing roughness. Perfectly flat, hydrophobic surfaces can only show water contact angles up to 130°. However, the presence of microstructures of suitable size is capable of creating superhydrophobicity. Such microstructures lead to the formation of air cavities on the surface which cannot be wetted by the water—the so-called Cassie-Baxter state. This leads to unexpected (from a chemical viewpoint) wetting phenomena as seen in nature, for example, in case of the lotus effect.

The ACA and RCA are displayed in Table 2 and compare well to values obtained by the SCA measurements (see Supplementary Materials, Figure S7) for the TMSC-coated substrates. Substrates with the lowest roughness show the lowest mean value between the ACA and RCA (θ_Si-TMSC_ < θ_PET-TMSC_ < θ_Glass-TMSC_ < θ_Al-TMSC_ < θ_Ni-TMSC_ < θ_Cu-TMSC_). The difference between the two values depends on the substrate hydrophobicity and roughness, while the superhydrophobic Cu surface has almost equal values for the ACA and RCA. 

In general, the dynamic WCAs for the substrates after the regeneration are higher than the static values (Supplementary Materials, Figures S8 and S9), ranging, except for the Cu sample, from 31° to 50°. As previously discussed, the formation of oxides and chlorides at the metal substrates influences the measurements. As visible in the SEM images after regeneration, there are no homogeneous films on the Cu and Al foils after regeneration, leading to contact angles close to the values of the bare metals. In contrast, on Ni, the cellulose film features typical values. For the other substrates, a rather strong CAH can be observed, giving almost the same difference between the ACA and RCA as observed for the TMSC-coated substrates. We observe the same trend, namely that increasing film roughness leads to larger CAH.

In the case of TMSC on copper, water droplets are rolling off from bent surfaces. Furthermore, if droplets are deposited from an approximately 5 cm height, they bounce back from the surface. This originates from the adhesion between the water molecules inside the droplet, which is stronger than the cohesion with the microstructured substrate; see Figure 4b. For these surfaces, there is an additional interesting feature: if a nonpolar liquid such as diiodomethane is placed on the surface, it completely spreads. This was previously reported for selected material classes, for example, high-impact polystyrene silica composites [46], organochlorosilanes [47], and copper surfaces modified with coatings of hydrophobic substances such as *n*-dodecylmercaptan [48]. Similar approaches have used silanized paper [49,50] and epoxy resins in combination with several silane components [51], to name some examples. Examples of superhydrophobic materials based on cellulose are rare and usually involve a second polymer to achieve microstructures via phase separation [52]. 

It is also noteworthy that after exposure to HCl vapors, the contact angles on the copper do not reach even hydrophilic regimes. Since even pronounced regeneration times did not change the contact angle significantly, there must a competing reaction between the metal oxide surface and the HCl taking place and preventing the formation of a smooth cellulose film. 

### 3.4. Surface Free Energy (SFE)

A valuable parameter to describe the surface properties of any type of material is the surface free energy. For solids, this can be simply performed using wettability testing with liquids differing in polarity. Different models such as the OWRK approach have been developed to connect the Young-Laplace equation used for the description of wetting phenomena to the surface free energy [31,33]. Although this concept is still under debate [53,54], mainly because fluorinated surfaces do not comply with the model, it is highly useful since it allows predicting the compatibility of different materials and allows for qualitative predictions of disperse and polar contributions to SFE. 

Due to the similarity of the CAH values and the SCA results, static contact angle measurements were performed to determine the SFE. The substrates chosen for the deposition of TMSC differ rather significantly in their SFE and range from 30 to nearly 80 mJ·m^−2^ (for details, see Supplementary Materials, Figure S10). In the cases of the more hydrophobic polymer and metal foils, the dispersive part is a significant contribution to the overall SFE. 

Coating the surfaces with TMSC changes the surface free energy completely, leading to surface free energies around 25 to 35 mJ·m^−2^ with hardly any polar contributions. The only exception is for the TMSC on the copper substrate, exhibiting significantly lower SFE (6 mJ·m^−2^), being in the range of a superhydrophobic material. After the exposure to HCl vapors, the SFE increased for all substrates, but only for some (glass, Si, PA, PET) values for cellulose (50 to 60 mJ·m^−2^) were reported. The values obtained from the metal foils are remarkably lower and less uniform. Undoubtedly, the generation of particles and the subsequent increase in roughness impacts the SFE in these determinations. As a consequence, the exposed surface may not be only composed of cellulose, but also of metal oxides/chlorides which blur the SFE. The only exception is copper, whose surface free energy still has a rather low value of 18 mJ·m^−2^ (compare: pure Cu: 60 mJ·m^−^², TMSC on Cu: 5 mJ·m^−^²).

## 4. Conclusions

It has been demonstrated that TMSC can be deposited on a variety of substrates with large size (up to 7.3 × 10 cm^2^) varying in surface free energy, polarity, stiffness, and roughness. The roughness and the SFE are identified as the most important factors, since they determine the film thickness and homogeneity of the formed coatings. Thus, homogeneous layers can be obtained for the PET and PA foils as well as for glass slides and silicon wafers, which have a low roughness and a high SFE. A peculiarity was the TMSC layer on the microstructured copper foil, where superhydrophobicity was observed. Water drops were observed to bounce on the substrates and roll off when the substrates were bent. 

Regeneration of TMSC using HCl vapors into cellulose is straightforward for PA, PET, glass, and silicon wafers, wherein the regeneration time correlates with the film thickness. The metal foils seem to be difficult substrates for regeneration, since they readily form metal chlorides/oxides at the TMSC–metal interface upon contact with gaseous HCl. Other acids must be employed which feature sufficient acidity to cleave off the TMS groups, but which do not attack the oxidized passivation layer present on the metal oxide. Trifluoroacetic acid could be a good candidate, which was proposed in a different type of regeneration procedure for cellulose xanthate [13].

## Figures and Tables

**Table 1:** Layer thickness and regeneration times on different substrates for the films deposited onto glass, silicon, PET, and PA substrates and metal foils.

Substrate	TMSC (nm)	Cellulose (nm)	Regen. Time (min)
**Glass^A^**	140 ± 1	50 ± 1	15
**Si^A^**	132 ± 1	46 ± 1	15
**PET^A^**	125 ± 5	43 ± 2	15
**PA^A^**	131 ± 7	44 ± 4	15
**Al^B^**	96 ± 16	n.d.	30
**Cu^B^**	545 ± 73	n.d.	30
**Ni^B^**	812 ± 29	n.d.	30

TMSC: ^A^ Determined by profilometry; ^B^ determined by cross-section analysis using SEM; n.d.: not determined.

**Table 2:** Water contact angle hysteresis of different substrates after the regeneration, starting at 6 µL drop size. The volume was slowly increased in steps of 0.5 µL up to a maximum of 17.5 µL, and then decreased over time with the same step size until a plateau was reached. All values are given in [°].

Film/Type of CA	Glass (°)	Si (°)	PET (°)	Cu (°)	Ni (°)	Al (°)
TMSC/ACA	100 ± 1	100 ± 1	99 ± 1	151 ± 1	107 ± 1	98 ± 1
TMSC/RCA	88 ± 1	80 ± 1	88 ± 1	149 ± 1	94 ± 1	90 ± 1
Cell ACA	51 ± 1	50 ± 1	44 ± 1	138 ± 1	33 ± 1	56 ± 1
Cell RCA	36 ± 1	31 ± 1	32 ± 1	105 ± 1	28 ± 1	44 ± 1

## Supplementary Materials

The following are available online at http://www.mdpi.com/1996-1944/11/12/2433/s1, Figure S1: IR spectra of blanks (black), TMSC coatings (red), and regenerated cellulose (blue) on A: Cu; B: Ni; and C: Al. The characteristic Si–C peaks are indicated by the black lines. Figure S2: Light microscopy of the Cu (top), Ni (middle), and Al (bottom) foils when blank (uncoated) (a), coated with TMSC (b), and after regeneration (c), respectively. Figure S3: Beam induced shrinkage of a TMSC film deposited on a nickel foil during SEM imaging with an electron energy of 3 keV. The shrinkage of 17% was observed within 10 s. Figure S4: SEM image (cross section) of a copper substrate coated with TMSC at a higher magnification. Figure S5: Effects of HCl vapor on TMSC films on Cu (top), Ni (middle), Al (bottom). The inhomogeneous layer at the aluminum foil is related to the oxidation of aluminum by HCl. Figure S6: Identification of the elemental composition of the TMSC layer on Cu, Ni and Al after exposure to HCl vapors by SEM (left) and corresponding analysis in the indicated area (rectangles or square) by EDX (right). Figure S7: Static water contact angles of TMSC coated substrates before (green) and after regeneration (blue) as well as the blank values (red lines). Figure S8: Water contact angle hysteresis at different substrates coated with TMSC starting at 6 µL drop size. The volume is slowly increased in steps of 0.5 µL up to 17.5 µL and then decreased over time with the same step size. Figure S9: Water contact angle hysteresis at different substrates after the regeneration starting at 6 µL drop size. The volume is slowly increased in steps of 0.5 µL up to 17.5 µL and then decreased over time with the same step size. Figure S10: Total surface free energy (red) with disperse (blue) and polar contribution (green) of blank substrates (left), TMSC (middle) and regenerated cellulose (right) respectively calculated via the OWRK method. In case of the blank copper foil the diiodomethane spreads too quick so for those contact angles of 5° are assumed for the calculations, thus no error bars for the filter paper blank can be determined.
