# Supplementary material for: Deposition of Cellulose-Based Thin Films on Flexible Substrates
Source: Materials (Basel). 2018 Nov 30;11(12):2433. doi: 10.3390/ma11122433 (PMC6316936; doi:10.3390/ma11122433)
Supplement: Supplementary file 1 [file materials-11-02433-s001.pdf]

Supplementary Materials: Deposition of Cellulose Based Thin Films on Flexible Substrates

Werner Schlemmer [1], Armin Zankel [2], Katrin Niegelhell [1], Mathias Hobisch [1], Michael Süssenbacher [1], Krisztina Zajki-Zechmeister [2], Michael Weissl [1], David Reishofer [1], Harald Plank [2] and Stefan Spirk [1,*]

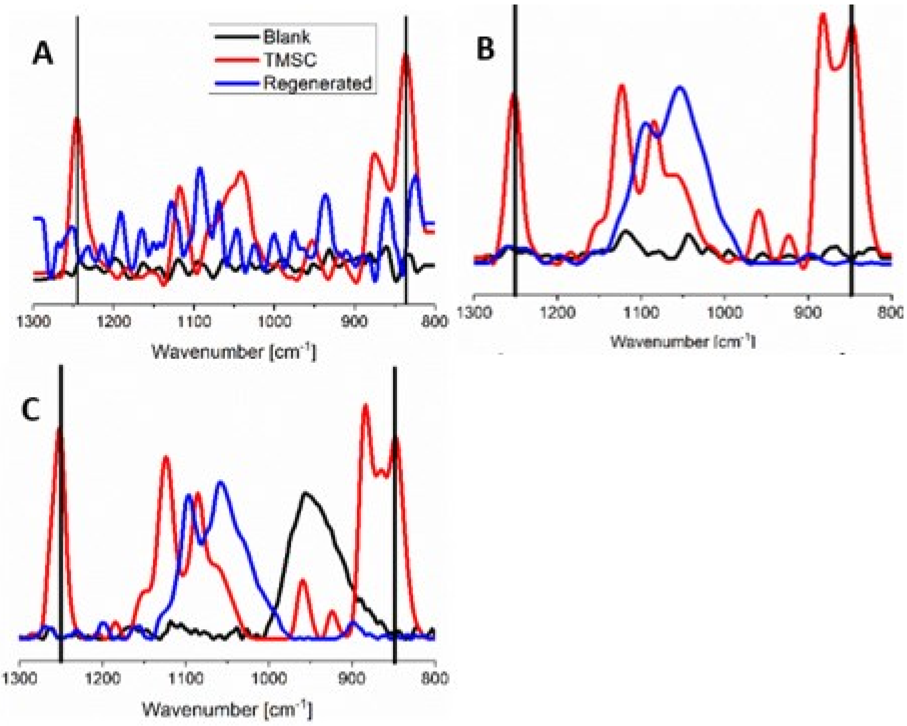

Figure S1. IR-Spectra of blanks (black), TMSC coatings (red) and regenerated cellulose (blue) on (**A**) Cu; (**B**) Ni; (**C**) Al. The characteristic Si–C peaks are indicated by the black lines.

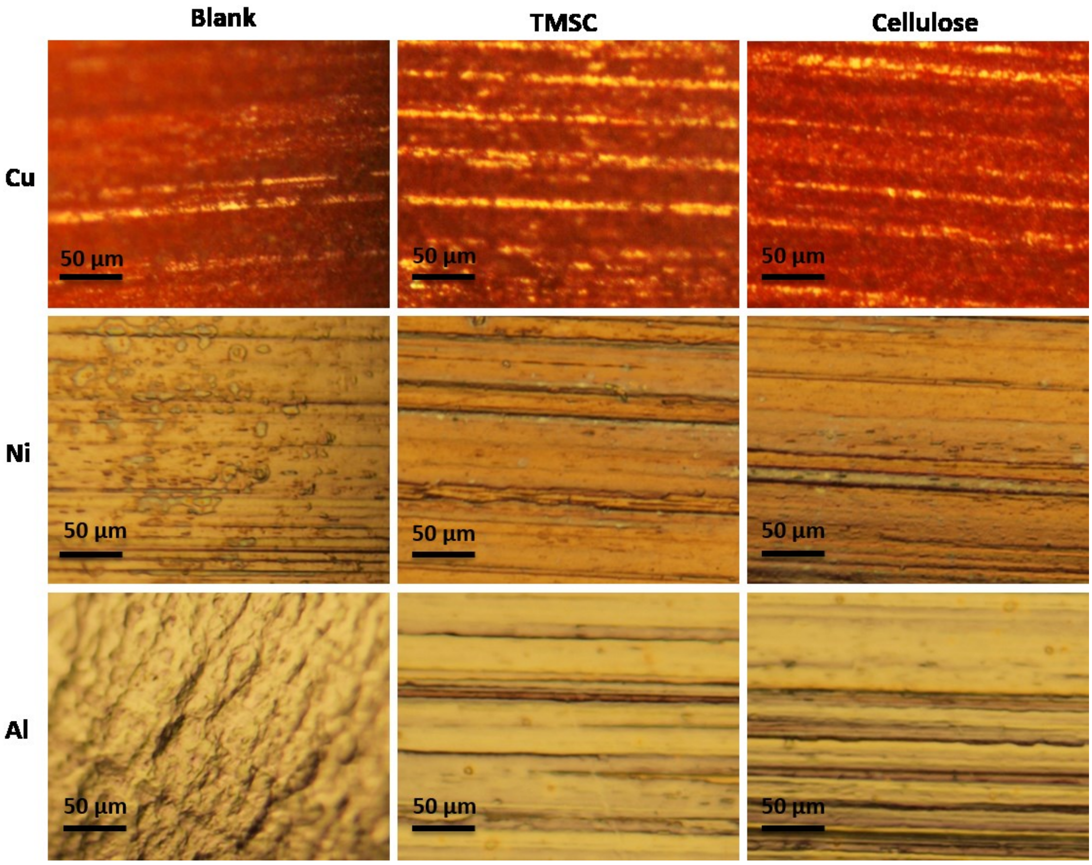

Figure S2. Light microscopy of the Cu (top), Ni (middle), and Al (bottom) foils blank (**a**), coated with TMSC (**b**) as well as after regeneration (**c**), respectively. The images were obtained using reflected illumination at a magnification of 1:500.

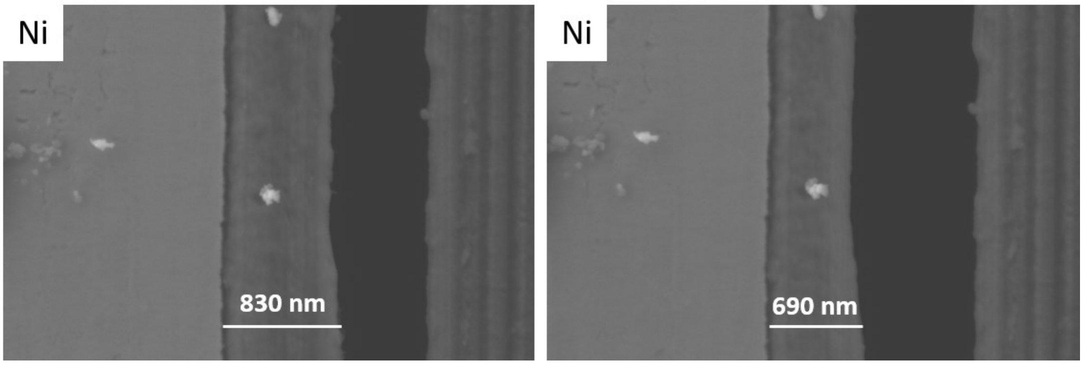

Figure S3. Beam induced shrinkage of a TMSC film deposited on a nickel foil during SEM imaging with an electron energy of 3 keV. The shrinkage of 17% was observed within 10 s.

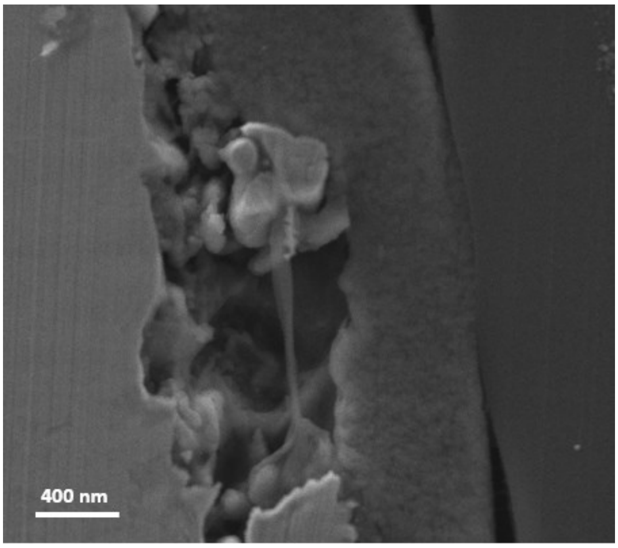

Figure S4. SEM image (cross section) of a copper substrate coated with TMSC at a higher magnification.

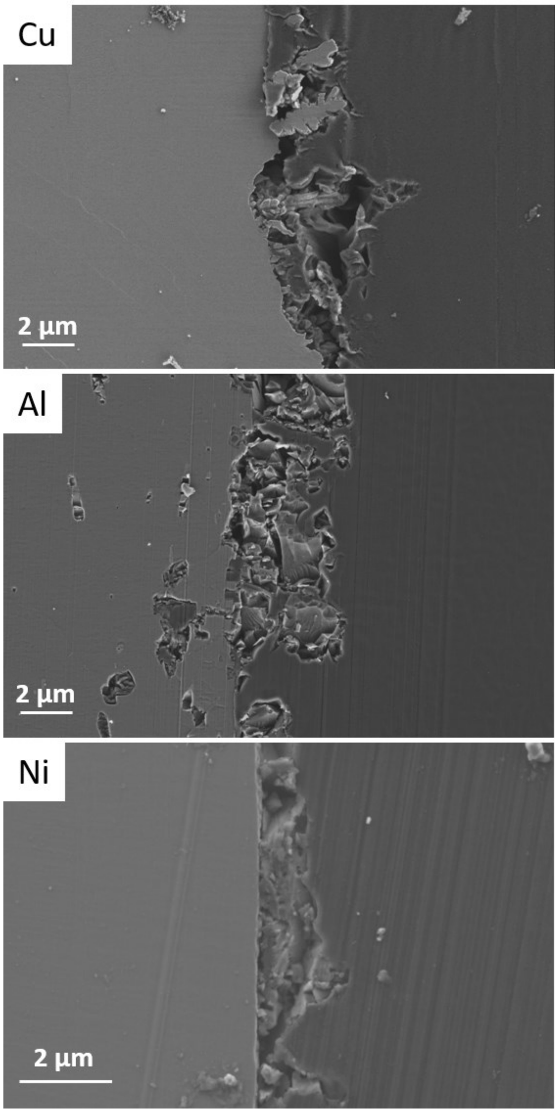

Figure S5. Effects of HCl vapor on TMSC films on Cu (top), Ni (middle), Al (bottom). The inhomogeneous layer at the aluminum foil is related to the oxidation of aluminum by HCl.

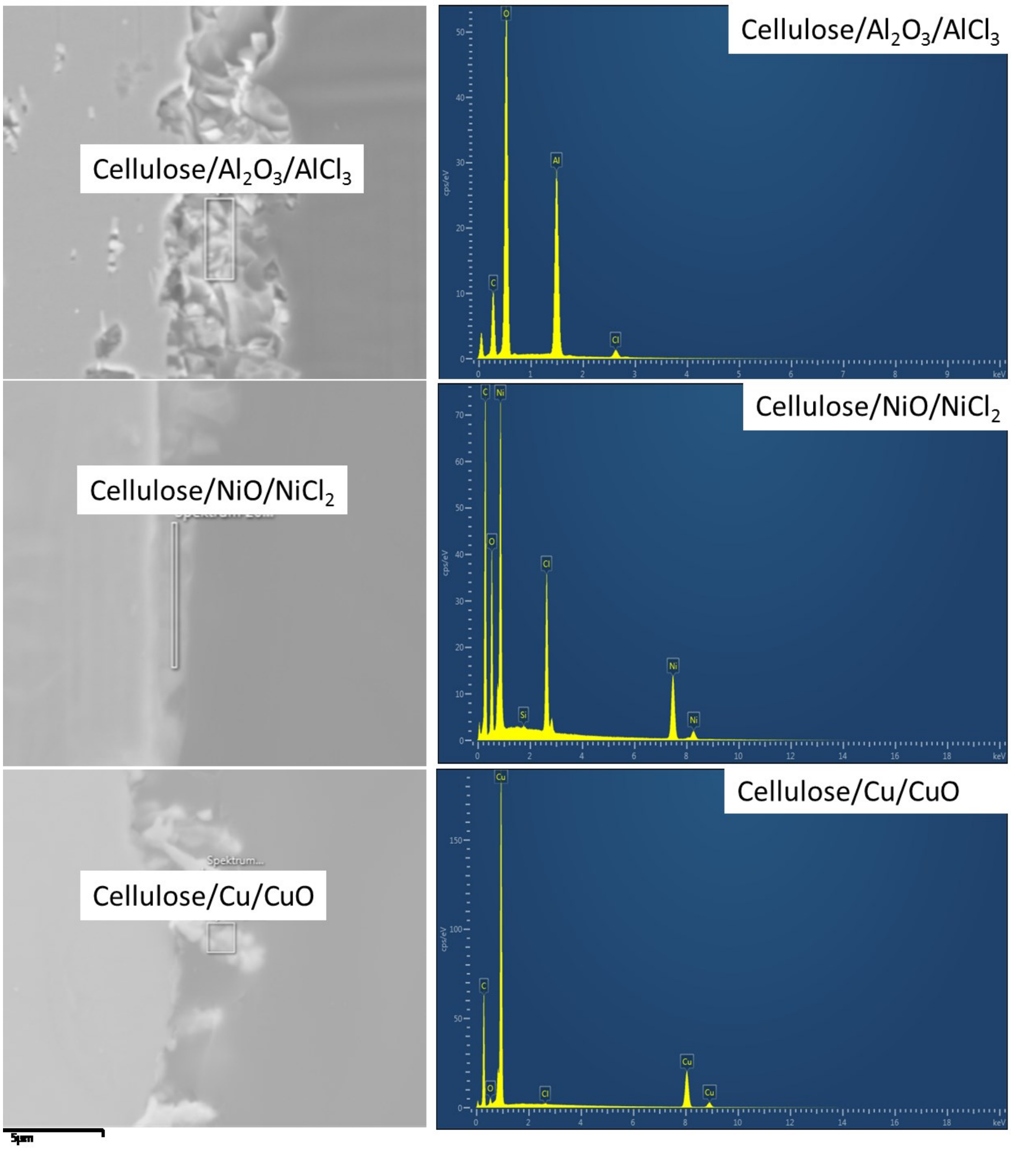

Figure S6. Identification of the elemental composition of the TMSC layer on Cu, Ni and Al after exposure to HCl vapors by SEM (left) and corresponding analysis in the indicated area (rectangles or square) by EDX (right).

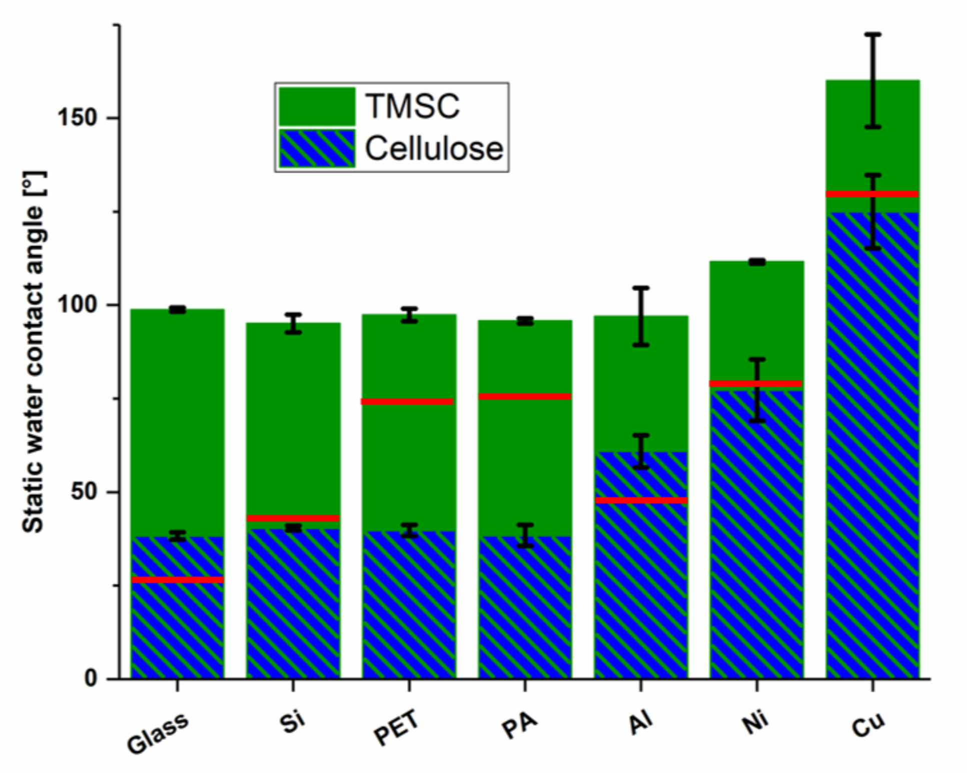

Figure S7. Static water contact angles of TMSC coated substrates before (green) and after regeneration (blue) as well as the blank values (red lines).

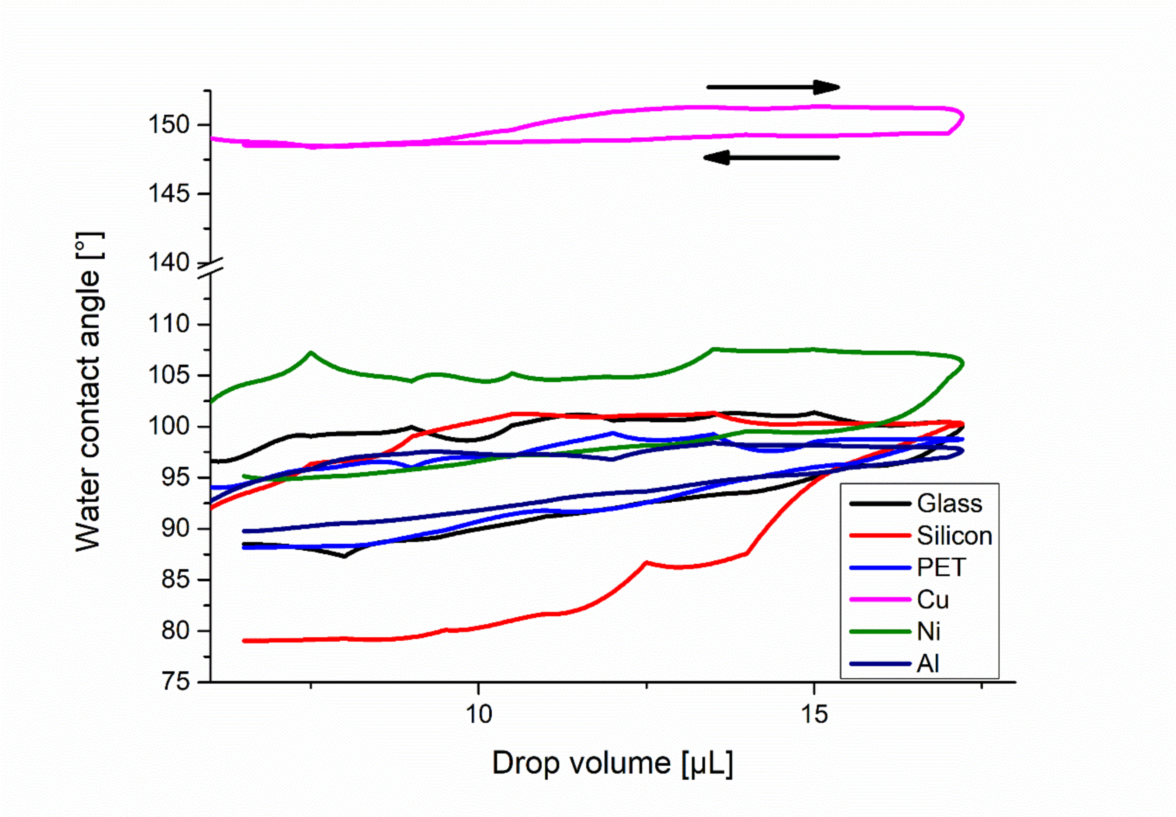

Figure S8. Water contact angle hysteresis at different substrates coated with TMSC starting at 6 µL drop size. The volume is slowly increased in steps of 0.5 µL up to 17.5 µL and then decreased over time with the same step size.

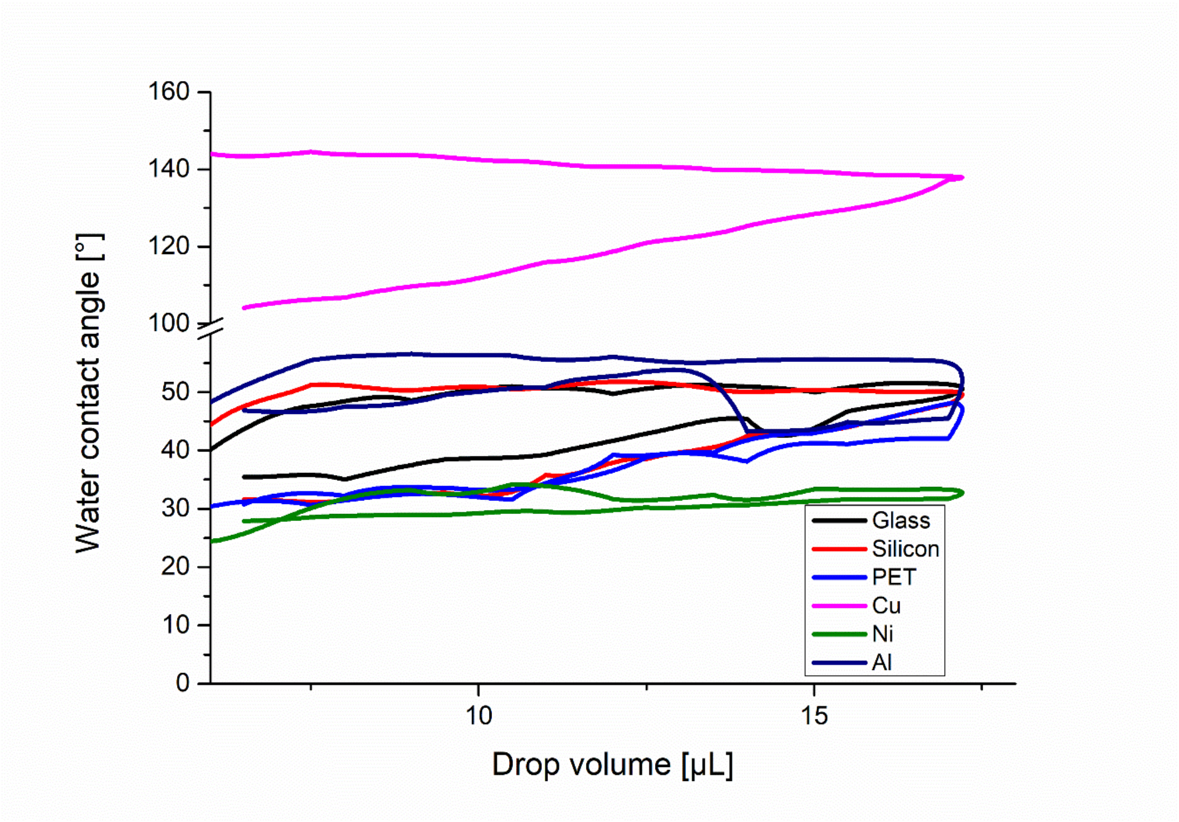

Figure S9. Water contact angle hysteresis at different substrates after the regeneration starting at 6 µL drop size. The volume is slowly increased in steps of 0.5 µL up to 17.5 µL and then decreased over time with the same step size.

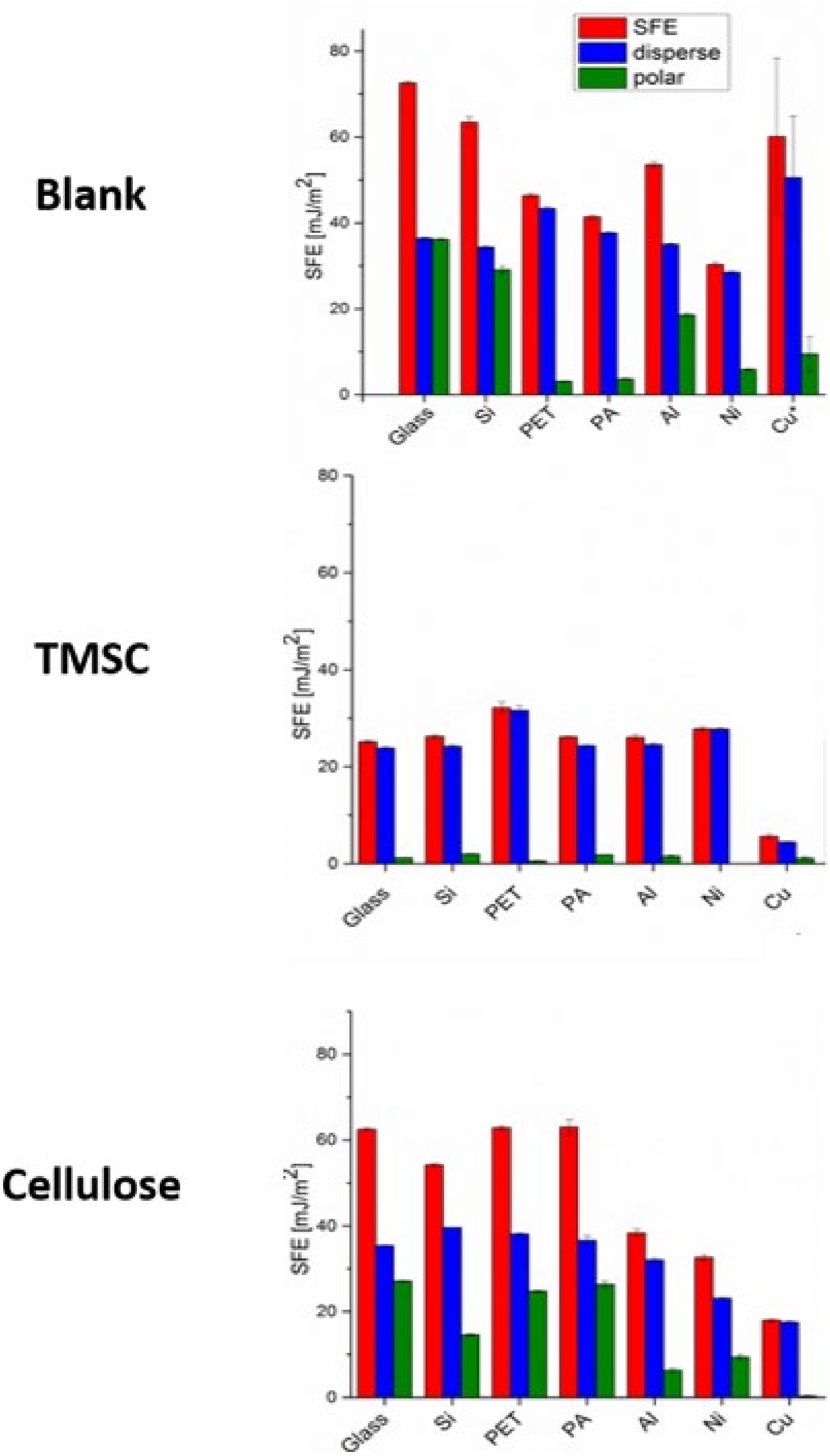

Figure S10. Total surface free energy (red) with disperse (blue) and polar contribution (green) of blank substrates (left), TMSC (middle) and regenerated cellulose (right) respectively calculated via the OWRK method. In case of the blank copper foil the diiodomethane spreads too quick so for those contact angles of 5° are assumed for the calculations, thus no error bars for the filter paper blank can be determined.
